# Graph convolutional networks: a comprehensive review

**DOI:** 10.1186/s40649-019-0069-y

**Published:** 2019-11-10

**Authors:** Si Zhang, Hanghang Tong, Jiejun Xu, Ross Maciejewski

**Affiliations:** 1 University of Illinois Urbana-Champaign, Champaign, USA; 2 HRL Laboratories, LLC, Malibu, USA; 3 Arizona State University, Tempe, USA

**Keywords:** Graph convolutional networks, Graph representation learning, Deep learning, Spectral methods, Spatial methods, Aggregation mechanism

## Abstract

Graphs naturally appear in numerous application domains, ranging from social analysis, bioinformatics to computer vision. The unique capability of graphs enables capturing the structural relations among data, and thus allows to harvest more insights compared to analyzing data in isolation. However, it is often very challenging to solve the learning problems on graphs, because (1) many types of data are not originally structured as graphs, such as images and text data, and (2) for graph-structured data, the underlying connectivity patterns are often complex and diverse. On the other hand, the representation learning has achieved great successes in many areas. Thereby, a potential solution is to learn the representation of graphs in a low-dimensional Euclidean space, such that the graph properties can be preserved. Although tremendous efforts have been made to address the graph representation learning problem, many of them still suffer from their shallow learning mechanisms. Deep learning models on graphs (e.g., graph neural networks) have recently emerged in machine learning and other related areas, and demonstrated the superior performance in various problems. In this survey, despite numerous types of graph neural networks, we conduct a comprehensive review specifically on the emerging field of graph convolutional networks, which is one of the most prominent graph deep learning models. First, we group the existing graph convolutional network models into two categories based on the types of convolutions and highlight some graph convolutional network models in details. Then, we categorize different graph convolutional networks according to the areas of their applications. Finally, we present several open challenges in this area and discuss potential directions for future research.

## Introduction

Graphs naturally arise in many real-world applications, including social analysis [1], fraud detection [2, 3], traffic prediction [4], computer vision [5], and many more. By representing the data as graphs, the structural information can be encoded to model the relations among entities, and furnish more promising insights underlying the data. For example, in a transportation network, nodes are often the sensors and edges represent the spatial proximity among sensors. In addition to the temporal information provided by the sensors themselves, the graph structure modeled by the spatial correlations leads to a prominent improvement in the traffic prediction problem [4]. Moreover, by modeling the transactions among people as a graph, the complex transaction patterns can be mined for synthetic identity detection [3] and money laundering detection [6].

However, the complex structure of graphs [7] often hampers the capability of gaining the true insights underlying the graphs. Such complexity, for example, resides in the non-Euclidean nature of the graph-structured data. A potential solution to dealing with the complex patterns is to learn the graph representations in a low-dimensional Euclidean space via embedding techniques, including the traditional graph embedding methods [8–10], and the recent network embedding methods [11, 12]. Once the low-dimensional representations are learned, many graph-related problems can be easily done, such as the classic node classification and link prediction [12]. There exist many thorough reviews on both traditional graph embedding and recent network embedding methods. For example, Yan et al. review several well-established traditional graph embedding methods and discuss the general framework for graph dimensionality reduction [13]. Hamilton et al. review the general graph representation learning methods, including node embedding and subgraph embedding [14]. Furthermore, Cui et al. discuss the differences between the traditional graph embedding and the recent network embedding methods [15]. One notable difference is that the recent network embedding is more suitable for the task-specific network inference. Other existing literature reviews on network embedding include [16, 17].

Despite some successes of these embedding methods, many of them suffer from the limitations of the shallow learning mechanisms [11, 12] and might fail to discover the more complex patterns behind the graphs. Deep learning models, on the other hand, have been demonstrated their power in many applications. For example, convolution neural networks (CNNs) achieve a promising performance in many computer vision [18] and natural language processing [19] applications. One key reason of such successes is that CNN models can highly exploit the stationarity and compositionality properties of certain types of data. In particular, due to the grid-like nature of images, the convolutional layers in CNNs enable to take advantages of the hierarchical patterns and extract high-level features of the images to achieve a great expressive capability. The basic CNN models aim to learn a set of fixed-size trainable localized filters which scan every pixel in the images and combine the surrounding pixels. The core components include the convolutional and pooling layers that can be operated on the data with an Euclidean or grid-like structure.

However, the non-Euclidean characteristic of graphs (e.g., the irregular structure) makes the convolutions and filtering on graphs not as well-defined as on images. In the past decades, researchers have been working on how to conduct convolutional operations on graphs. One main research direction is to define graph convolutions from the spectral perspective, and thus, graph signal processing, such as graph filtering and graph wavelets, has attracted lots of research interests. Shuman et al. give a comprehensive overview of graph signal processing, including the common operations and analyses on graphs [20]. Briefly speaking, spectral graph convolutions are defined in the spectral domain based on graph Fourier transform, an analogy of 1-D signal Fourier transform. In this way, the spectral-based graph convolutions can be computed by taking the inverse Fourier transform of the multiplication between two Fourier transformed graph signals. On the other hand, graph convolution can be also defined in the spatial domain (i.e., vertex domain) as the aggregations of node representations from the node neighborhoods. The emergence of these operations opens a door to graph convolutional networks. Generally speaking, graph convolutional network models are a type of neural network architectures that can leverage the graph structure and aggregate node information from the neighborhoods in a convolutional fashion. Graph convolutional networks have a great expressive power to learn the graph representations and have achieved a superior performance in a wide range of tasks and applications. Note that in the past few years, many other types of graph neural networks have been proposed, including (but are not limited to): (1) graph auto-encoder [21], (2) graph generative model [22, 23], (3) graph attention model [24, 25], and (4) graph recurrent neural networks [26, 27].

**Fig. 1:**
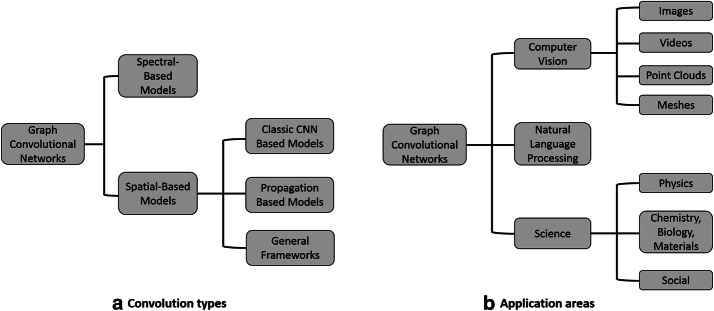
An overview of graph convolutional networks

There exist several other related surveys on the topic of graph neural networks. Bronstein et al. review the mathematical details and a number of early approaches of geometric deep learning for both graphs and manifolds [28]. Zhang et al. present a detailed review that covers many existing graph neural networks beyond graph convolutional networks, such as graph attention networks and gated graph neural network [29]. In addition, Wu et al. also review the studies on graph generative models and neural networks for spatial-temporal networks [30]. Besides, Lee et al. present an overview of graph neural networks with a special focus on graph attention networks [31]. However, since graph convolutional network is a very hot and fast developing research area, these existing surveys may not cover the most up-to-date models. In this survey, we focus specifically on reviewing the existing literature of the graph convolutional networks and cover the recent progress. The main contributions of this survey are summarized as follows:

1. We introduce two taxonomies to group the existing graph convolutional network models (Fig. 1). First, we categorize graph convolutional networks into spectral-based and spatial-based models depending on the types of convolutions. Then, we introduce several graph convolutional networks according to their application domains.
2. We motivate each taxonomy by surveying and discussing the up-to-date graph convolutional network models.
3. We discuss the challenges of the current models that need to be addressed and highlight some promising directions for the future work.

The rest of the paper is organized as follows. We start by summarizing the notations and introducing some preliminaries of graph convolutional networks in “Notations and preliminaries” section. Then, in “Spectral graph convolutional networks” and “Spatial graph convolutional networks” sections, we categorize the existing models into the spectral-based methods and the spatial-based methods by the types of graph filtering with some detailed examples. “Applications of graph convolutional networks” section presents the methods from a view of applications. In “Challenges and future researches” section, we discuss some challenges of the existing graph convolutional network models and provide some directions for the future work. Finally, we conclude our survey in “Concluding remarks” section.

## Notations and preliminaries

In this section, we present the notations and some preliminaries for the graph convolutional networks. In general, we use bold uppercase letters for matrices, bold lowercase letters for vectors, and lowercase letters for scalars. For matrix indexing, we use $\mathbf{A}(i,j)$ to denote the entry at the intersection of the *i*th row and *j*th column. We denote the transpose of a matrix $\mathbf{A}$ as $\mathbf{A}^T$.

### Graphs and graph signals

In this survey, we are interested in the graph convolutional network models on an undirected connected graph $\mathcal {G}=\{\mathcal {V},\mathcal {E},\mathbf{A}\}$, which consists of a set of nodes $\mathcal {V}$ with $|\mathcal {V}|=n$, a set of edges $\mathcal {E}$ with $|\mathcal {E}|=m$ and the adjacency matrix $\mathbf{A}$. If there is an edge between node *i* and node *j*, the entry $\mathbf{A}(i,j)$ denotes the weight of the edge; otherwise, $\mathbf{A}(i,j)=0$. For unweighted graphs, we simply set $\mathbf{A}(i,j)=1$. We denote the degree matrix of $\mathbf{A}$ as a diagonal matrix ${\mathbf{D}}$, where ${\mathbf{D}}(i,i)=\sum _{j=1}^{n}\mathbf{A}(i,j)$. Then, the Laplacian matrix of $\mathbf{A}$ is denoted as ${\mathbf{L}}={\mathbf{D}}-\mathbf{A}$. The corresponding symmetrically normalized Laplacian matrix is $\tilde{{\mathbf{L}}}=\mathbf{I}-{\mathbf{D}}^{-\frac{1}{2}}\mathbf{A}{\mathbf{D}}^{-\frac{1}{2}}$, where $\mathbf{I}$ is an identity matrix.

A graph signal defined on the nodes is represented as a vector ${\mathbf{x}}\in \mathbb {R}^n$, where ${\mathbf{x}}(i)$ is the signal value on the node *i* [20]. Node attributes, for instance, can be considered as the graph signals. Denote $\mathbf{X}\in \mathbb {R}^{n\times d}$ as the node attribute matrix of an attributed graph, and then, the columns of $\mathbf{X}$ are the *d* signals of the graph.

### Graph Fourier transform

It is well-known that the classic Fourier transform of an 1-D signal *f* is computed by $\hat{f}(\xi )=\langle f,e^{2\pi i\xi t}\rangle$, where $\xi$ is the frequency of $\hat{f}$ in the spectral domain and the complex exponential is the eigenfunction of the Laplace operator. Analogously, the graph Laplacian matrix ${\mathbf{L}}$ is the Laplace operator defined on a graph. Hence, an eigenvector of ${\mathbf{L}}$ associated with its corresponding eigenvalue is an analog to the complex exponential at a certain frequency. Note that the symmetrically normalized Laplacian matrix $\tilde{{\mathbf{L}}}$ and the random-walk transition matrix can be also used as the graph Laplace operator. In particular, denote the eigenvalue decomposition of $\tilde{{\mathbf{L}}}$ as $\tilde{{\mathbf{L}}}=\mathbf{U}\varvec{\Lambda }\mathbf{U}^T$ where the *l*th column of $\mathbf{U}$ is the eigenvector $\mathbf{u}_l$ and $\varvec{\Lambda }(l,l)$ is the corresponding eigenvalue $\lambda _l$, and then, we can compute the Fourier transform of a graph signal ${\mathbf{x}}$ as:

1 $$\begin{aligned} \hat{{\mathbf{x}}}(\lambda _l)=\langle {\mathbf{x}},\mathbf{u}_l\rangle =\sum _{i=1}^{n}{\mathbf{x}}(i)\mathbf{u}^*_l(i). \end{aligned}$$

The above equation represents in the spectral domain a graph signal defined in the vertex domain. Then, the inverse graph Fourier transform can be written as:

2 $$\begin{aligned} {\mathbf{x}}(i)=\sum _{l=1}^{n}\hat{{\mathbf{x}}}(\lambda _l)\mathbf{u}_l(i). \end{aligned}$$

### Graph filtering

Graph filtering is a localized operation on graph signals. Analogous to the classic signal filtering in the time or spectral domain, one can localize a graph signal in its vertex domain or spectral domain, as well.

(1) Frequency filtering: Recall that the frequency filtering of a classic signal is often represented as the convolution with the filter signal in the time domain. However, due to the irregular structure of the graphs (e.g., different nodes having different numbers of neighbors), graph convolution in the vertex domain is not as straightforward as the classic signal convolution in the time domain. Note that for classic signals, the convolution in the time domain is equivalent to the inverse Fourier transform of the multiplication between the spectral representations of two signals. Therefore, the spectral graph convolution is defined analogously as:

3 $$\begin{aligned} ({\mathbf{x}}*_{\mathcal {G}}\mathbf{y})(i)=\sum _{l=1}^{n}\hat{{\mathbf{x}}}(\lambda _l)\hat{\mathbf{y}}(\lambda _l)\mathbf{u}_l(i). \end{aligned}$$

Note that $\hat{{\mathbf{x}}}(\lambda _l)\hat{\mathbf{y}}(\lambda _l)$ indicates the filtering in the spectral domain. Thus, the frequency filtering of a signal ${\mathbf{x}}$ on graph $\mathcal {G}$ with a filter $\mathbf{y}$ is exactly same as Eq. (3) and is further re-written as:

4 $$\begin{aligned} {\mathbf{x}}_{out}={\mathbf{x}}*_{\mathcal {G}}\mathbf{y}=\mathbf{U} \begin{bmatrix} \hat{\mathbf{y}}(\lambda _1)&0 \\&\ddots&\\ 0&\hat{\mathbf{y}}(\lambda _n) \end{bmatrix} \mathbf{U}^T{\mathbf{x}}. \end{aligned}$$

(2) Vertex filtering: The graph filtering of a signal ${\mathbf{x}}$ in the vertex domain is generally defined as a linear combination of the signal components in the nodes neighborhood. Mathematically, the vertex filtering of a signal ${\mathbf{x}}$ at node *i* is:

5 $$\begin{aligned} {\mathbf{x}}_{out}(i)=w_{i,i}{\mathbf{x}}(i)+\sum _{j\in \mathcal {N}(i,K)}w_{i,j}{\mathbf{x}}(j), \end{aligned}$$

where $\mathcal {N}(i,K)$ represents the *K*-hop neighborhood of node *i* in the graph and the parameters $\{w_{i,j}\}$ are the weights used for the combination. It can be shown that using a *K*-polynomial filter, the frequency filtering can be interpreted from the vertex filtering perspective [20].

## Spectral graph convolutional networks

In this section and the subsequent “Spatial graph convolutional networks” section, we categorize the graph convolutional neural networks into the spectral-based methods and the spatial-based methods, respectively. We consider the spectral-based methods to be those methods that start with constructing the frequency filtering.

The first notable spectral-based graph convolutional network is proposed by Bruna et al. [32]. Motivated by the classic CNN, this deep model on graphs contains several spectral convolutional layers that take a vector $\mathbf{X}^p$ of size $n\times d_{p}$ as the input feature map of the *p*th layer and output a feature map $\mathbf{X}^{p+1}$ of size $n\times d_{p+1}$ by:

6 $$\begin{aligned} \mathbf{X}^{p+1}(:, j)=\sigma \left( \sum _{i=1}^{d_p}\mathbf{V} \begin{bmatrix} (\varvec{\theta }_{i,j}^p)(1)& 0 \\& \ddots& \\ 0& (\varvec{\theta }_{i,j}^p)(n) \end{bmatrix} \mathbf{V}^T\mathbf{X}^p(:,i)\right), \quad \forall j=1,\cdots ,d_{p+1}, \end{aligned}$$

where $\mathbf{X}^p(:,i)$ ($\mathbf{X}^{p+1}(:,j)$) is the *i*th (*j*th) dimension of the input (output) feature map, respectively; $\varvec{\theta }_{i,j}^p$ denotes a vector of learnable parameters of the filter at the *p*th layers. Each column of $\mathbf{V}$ is the eigenvector of ${\mathbf{L}}$ and $\sigma (\cdot )$ is the activation function. However, there are several issues with this convolutional structure. First, the eigenvector matrix $\mathbf{V}$ requires the explicit computation of the eigenvalue decomposition of the graph Laplacian matrix, and hence suffers from the $O(n^3)$ time complexity which is impractical for large-scale graphs. Second, though the eigenvectors can be pre-computed, the time complexity of Eq. (6) is still $O(n^2)$. Third, there are *O*(*n*) parameters to be learned in each layer. Besides, these non-parametric filters are not localized in the vertex domain. To overcome the limitations, the authors also propose to use a rank-*r* approximation of eigenvalue decomposition. To be specific, they use the first *r* eigenvectors of $\mathbf{V}$ that carry the most smooth geometry of the graph and consequently reduce the number of parameters of each filter to *O*(1). Moreover, if the graph contains the clustering structure that can be explored via such a rank-*r* factorization, the filters are potentially localized. Building upon [32], Henaff et al. propose to apply an input smoothing kernel (e.g., splines) and use the corresponding interpolated weights as the filter parameters for graph spectral convolutions [33]. As claimed in [33], the spatial localization in the vertex domain can be somewhat achieved. However, the computational complexity and the localization power still hinder learning better representations of the graphs.

To address these limitations, Defferrard et al. propose the ChebNet that uses *K*-polynomial filters in the convolutional layers for localization [34]. Such a *K*-polynomial filter is represented by $\hat{\mathbf{y}}(\lambda _l)=\sum _{k=1}^{K}\theta _k\lambda _l^k$. As mentioned in “Notations and preliminaries” section, the *K*-polynomial filters achieve a good localization in the vertex domain by integrating the node features within the *K* hop neighborhood [20], and the number of the trainable parameters decreases to $O(K)=O(1)$. In addition, to further reduce the computational complexity, the Chebyshev polynomial approximation [35] is used to compute the spectral graph convolution. Mathematically, the Chebyshev polynomial $T_k(x)$ of order *k* can be recursively computed by $T_k(x)=2xT_{k-1}(x)-T_{k-2}(x)$ with $T_0=1,~T_1(x)=x$. Defferrard et al. normalize the filters by $\tilde{\lambda }_l=2\frac{\lambda _l}{\lambda _{\text {max}}}-1$ to make the scaled eigenvalues lie within $[-1, 1]$. As a result, the convolutional layer is:

7 $$\begin{aligned} \mathbf{X}^{p+1}(:,j)=\sigma \left( \sum _{i=1}^{d_p}\sum _{k=0}^{K-1} (\varvec{\theta }_{i,j}^p)(k+1)T_k(\tilde{{\mathbf{L}}})\mathbf{X}^p(:,i)\right) , \quad \forall j=1,\ldots ,d_{p+1}, \end{aligned}$$

where $\varvec{\theta }_{i,j}^p$ is a *K*-dimensional parameter vector for the *i*th column of input feature map and the *j*th column of output feature map at the $-p$th layer. The authors also design a max-pooling operation with the multilevel clustering method Graclus [36] which is quite efficient to uncover the hierarchical structure of the graphs.

As a special variant, the graph convolutional network proposed by Kipf et al. (named as GCN) aims at the semi-supervised node classification task on graphs [37]. In this model, the authors truncate the Chebyshev polynomial to first-order (i.e., $K=2$ in Eq. (7)) and specifically set $(\varvec{\theta })_{i,j}(1)=-(\varvec{\theta })_{i,j}(2)=\theta _{i,j}$. Besides, since the eigenvalues of $\tilde{{\mathbf{L}}}$ are within [0, 2], relaxing $\lambda _{\text {max}}=2$ still guarantees $-1\le \tilde{\lambda }_l\le 1,~\forall l=1,\cdots ,n$. This leads to the simplified convolution layer as:

8 $$\begin{aligned} \mathbf{X}^{p+1}=\sigma \left( \tilde{{\mathbf{D}}}^{-\frac{1}{2}}\tilde{\mathbf{A}}\tilde{{\mathbf{D}}}^{-\frac{1}{2}}\mathbf{X}^p\varvec{\Theta }^p\right), \end{aligned}$$

where $\tilde{\mathbf{A}}=\mathbf{I}+\mathbf{A}$ is equivalent to adding self-loops to the original graph and $\tilde{{\mathbf{D}}}$ is the diagonal degree matrix of $\tilde{\mathbf{A}}$, and $\varvec{\Theta }^p$ is a $d_{p+1}\times d_p$ parameter matrix. Besides, Eq. (8) has a close relationship with the Weisfeiler–Lehman isomorphism test [38]. In addition, since Eq. (8) is essentially equivalent to aggregating node representations from their direct neighborhood, GCN has a clear meaning of vertex localization and, thus, is often considered as bridging the gap between the spectral-based methods and spatial-based methods. However, the training process could be costly in terms of memory for large-scale graphs. Moreover, the transduction of GCN interferes with the generalization, making the learning of representations of the unseen nodes in the same graph and the nodes in an entirely different graph more difficult [37].

To address the issues of GCN [37], FastGCN [39] improves the original GCN model by enabling the efficient minibatch training. It first assumes that the input graph $\mathcal {G}$ is an induced subgraph of a possibly infinite graph $\mathcal {G}'$, such that the nodes $\mathcal {V}$ of $\mathcal {G}$ are i.i.d. samples of the nodes of $\mathcal {G}'$ (denoted as $\mathcal {V}'$) under some probability measure $\mathcal {P}$. This way, the original convolution layer represented by Eq. (8) can be approximated by Monte Carlo sampling. Denote some i.i.d. samples $u_1^p,\ldots ,u_{t_p}^p$ at layer-*p*, the graph convolution can be estimated by:

9 $$\begin{aligned} \mathbf{X}^{p+1}(v,:)=\sigma \left( \frac{1}{t_p}\sum _{i=1}^{t_p}\tilde{\mathbf{A}}(v,u_i^p)\mathbf{X}^p(u_i^p,:)\varvec{\Theta }^p\right). \end{aligned}$$

Note that this Monte Carlo estimator of graph convolution could lead to a high variance of estimation. To reduce the variance, the authors formulate the variance and solve for an importance sampling distribution $\mathcal {P}$ of nodes. In addition, Chen et al. develop control variate-based algorithms to approximate GCN model [37] and propose an efficient sampling-based stochastic algorithm for training [40]. Besides, the authors theoretically prove the convergence of the algorithm regardless of the sampling size in the training phase [40]. Recently, Huang et al. develop an adaptive layer-wise sampling method to accelerate the training process in GCN models [41]. They first construct the layers in a graph convolutional network in a top-down way and then propose a layer-wise sampler to avoid the over-expansion of the neighborhoods due to the fixed-size sampling. To further reduce the variance, an explicit importance sampling is derived.

In parallel to the above models built upon Chebyshev polynomial approximations, other localized polynomial filters and their corresponding graph convolutional network models have also been proposed. For example, Levie et al. propose to use a more complex approximation method, namely Cayley polynomial, to approximate filters [42]. The proposed CayleyNet model is motivated by the fact that as the eigenvalues of the Laplacian matrix used in Chebyshev polynomials are scaled to the band $[-1, 1]$, the narrow frequency bands (i.e., eigenvalues concentrated around one frequency) are hard to be detected. Given that this narrow-band characteristic often appears in the community-structured graphs, ChebNet has limited flexibility and performance in a broader range of graph mining problems. Specifically, the Cayley filters of order K have the following form:^1^

10 $$\begin{aligned} \hat{\mathbf{y}}_{{\mathbf{c}},h}(\lambda _l)=c_0+2\text {Re}\left\{ \sum _{k=1}^{K} c_k(h\lambda _l-i)^j(h\lambda _l+i)^{-j}\right\}, \end{aligned}$$

where ${\mathbf{c}}=[c_0,\cdots ,c_K]$ are the parameters to be learned and $h>0$ is a spectral zoom parameter used to dilate graph spectrum, so that the Cayley filters can specialize different frequency bands. The localization property as well as the linear complexity can be achieved by further using Jacobi approximation [42]. In addition, LanczosNet [43] is proposed to encode the multi-scale characteristic naturally resided in graphs and penetrates the computation bottleneck of most existing models that involve the exponentiated graph Laplacian in the graph convolution operators to capture multi-scale information (e.g., [34]). In detail, the authors first compute the low rank approximation of matrix $\tilde{\mathbf{A}}$ by Lanczos algorithm, such that $\tilde{\mathbf{A}}\approx \mathbf{V}\mathbf{R} \mathbf{V}^T$, where $\mathbf{V}=\mathbf{Q}\mathbf{B}$, $\mathbf{Q}\in \mathbb {R}^{n\times K}$ contains the first *K* Lanczos vectors, and $\mathbf{B}\mathbf{R}\mathbf{B}^T$ is the eigen-decomposition of a tridiagonal matrix $\mathbf{T}$. In this way, the *t*th power of $\tilde{\mathbf{A}}$ can be simply approximated by $\tilde{\mathbf{A}}^t\approx \mathbf{V}\mathbf{R}^t\mathbf{V}^T$. Based on this, the proposed spectral filter in LanczosNet is formulated as:

11 $$\begin{aligned} \mathbf{X}^{p+1}(:,j)=[\mathbf{X}^p(:,i),\mathbf{V}\hat{\mathbf{R}}(1)\mathbf{V}^T\mathbf{X}^p(:,i), \ldots ,\mathbf{V}\hat{\mathbf{R}}(K-1)\mathbf{V}^T\mathbf{X}^p(:,i)]\varvec{\Theta }_{i,j}, \end{aligned}$$

where $\hat{\mathbf{R}}(k)=f_k([\mathbf{R}^0,\ldots ,\mathbf{R}^{K-1}])$ is a diagonal matrix and $f_k$ is a multi-layer perceptron (MLP). To leverage the multi-scale information, the above spectral filter is modified by adding short-scale parameters and long-scale parameters. A variant for node representation learning is also proposed in [43]. Beyond the Fourier transform-based spectral filters, Xu et al. propose to use the spectral wavelet transform on graphs, such that the consequent model can vary different scales of graphs to be captured [44].

Moreover, since many graph structures are manually constructed based upon the similarities among data points (e.g., kNN graphs), these fixed graphs may not have the best learning capability for some specific tasks. To this end, Li et al. propose a spectral graph convolution layer that can simultaneously learn the graph Laplacian [45]. In particular, instead of directly parameterizing the filter coefficients, the spectral graph convolution layer parameterizes a function over the graph Laplacian by introducing a notion of residual Laplacian. However, the main drawback of this method is the inevitable $O(n^2)$ complexity.

## Spatial graph convolutional networks

As the spectral graph convolution relies on the specific eigenfunctions of Laplacian matrix, it is still nontrivial to transfer the spectral-based graph convolutional network models learned on one graph to another graph whose eigenfunctions are different. On the other hand, according to the graph filtering in vertex domain (i.e., Eq. (5)), graph convolution can be alternatively generalized to some aggregations of graph signals within the node neighborhood. In this section, we categorize the spatial graph convolutional networks into the classic CNN-based models, propagation-based models, and other related general frameworks.

### Classic CNN-based spatial graph convolutional networks

Classic CNN models on grid-like data, such as images, have been shown great successes in many related applications, including images classification [46–48], object detection [18, 49], semantic segmentation [50, 51], etc. The basic properties of grid-like data that are exploited by convolution architectures include: (1) the number of neighboring pixels for each pixel is fixed, and (2) the spatial order of scanning images is naturally determined, i.e., from left to right and from top to bottom. However, different from images, neither the number of neighboring units nor the spatial order among them is fixed in the arbitrary graph data.

To address these issues, many works have been proposed to build graph convolutional networks directly upon the classic CNNs. Niepert et al. propose to address the aforementioned challenges by extracting locally connected regions from graphs [52]. The proposed PATCHY-SAN model first determines the nodes ordering by a given graph labeling approach such as centrality-based methods (e.g., degree, PageRank, betweenness, etc.) and selects a fixed-length sequence of nodes. Second, to address the issue of arbitrary neighborhood size of nodes, a fixed-size neighborhood for each node is constructed. Finally, the neighborhood graph is normalized according to graph labeling procedures, so that nodes of similar structural roles are assigned similar relative positions, followed by the representation learning with classic CNNs. However, as the spatial order of nodes is determined by the given graph labeling approach that is often solely based on graph structure, PATCHY-SAN lacks the learning flexibility and generality to a broader range of applications.

Different from PATCHY-SAN that order nodes by structural information [52], LGCN model [53] is proposed to transform the irregular graph data to grid-like data by using both structural information and input feature map of the *p*-th layer. In particular, for a node $u\in \mathcal {V}$ in $\mathcal {G}$, it stacks the input feature map of the node *u*’s neighbors into a single matrix $\mathbf{M}\in \mathbb {R}^{|\mathcal {N}(u)|\times d_p}$, where $|\mathcal {N}(u)|$ represents the number of 1-hop neighboring nodes of node *u*. For each column of $\mathbf{M}$, the first *r* largest values are preserved and form a new matrix $\tilde{\mathbf{M}}\in \mathbb {R}^{r\times d_p}$. In such a simple way, the input feature map along with the structural information of the graph can be transformed to an 1-D grid-like data $\tilde{\mathbf{X}}_p\in \mathbb {R}^{n\times (r+1)\times d_p}$. Then, the classic 1-D CNN can be applied to $\tilde{\mathbf{X}}^p$ and learn new node representations $\mathbf{X}^{p+1}$. Note that a subgraph-based training method is also proposed to scale the model to large-scale graphs.

As the convolution in the classic CNNs can only manage the data with the same topological structures, another way to extend the classic CNNs to graph data is to develop a structure-aware convolution operation for both Euclidean and non-Euclidean data. Chang et al. first build the connection between the classical filters and univariate functions (i.e., functional filters) and then model the graph structure into the generalized functional filters to be structural aware [54]. Since this structure-aware convolution requires infinite parameters to be learned, the Chebyshev polynomial [35] is used for approximation. Another work [55] re-architects the classic CNN by designing a set of fixed-size learnable filters (e.g., size-1 up to size-K) and shows that these filters are adaptive to the topology of the graph.

### Propagation-based spatial graph convolutional networks

In this subsection, we focus on the spatial graph convolutions that propagate and aggregate the node representations from neighboring nodes in the vertex domain. One notable work is [56] where the graph convolution for node *u* at the *p*th layer is designed as:

12 $$\begin{aligned} {\mathbf{x}}_{\mathcal {N}(u)}^p&=\mathbf{X}^p(u,:)+\sum _{v\in \mathcal {N}(u)} \mathbf{X}^p(v,:) \end{aligned}$$

13 $$\begin{aligned} \mathbf{X}^{p+1}(u,:) =\sigma \left({\mathbf{x}}_{\mathcal {N}(u)}^p \varvec{\Theta }_{|\mathcal {N}(u)|}^p\right), \end{aligned}$$

where $\varvec{\Theta }_{|\mathcal {N}(u)|}^p$ is the weight matrix for nodes with the same degree as $|\mathcal {N}(u)|$ at the *p*-=th layer. However, for arbitrarily large graphs, the number of unique values of node degree is often a very large number. Consequently, there will be many weight matrices to be learned at each layer, possibly leading to the overfitting problem.

Atwood et al. propose a diffusion-based graph convolutional network (named as DCNN) which evokes the propagations and aggregations of node representations by graph diffusion processes [57]. A *k*-step diffusion is conducted by the *k*th power of transition matrix ${\mathbf{P}}^k$, where ${\mathbf{P}}={\mathbf{D}}^{-1}\mathbf{A}$. Then, the diffusion–convolution operation is formulated as:

14 $$\begin{aligned} {\mathbf{Z}}(u,k,i)=\sigma \left( \varvec{\Theta }(k,i)\sum _{v=1}^{n} {\mathbf{P}}^k(u,v)\mathbf{X}(v,i)\right), \end{aligned}$$

where ${\mathbf{Z}}(u,k,i)$ is the *i*th output feature of node *u* aggregated based on ${\mathbf{P}}^k$ and the nonlinear activation function $\sigma (\cdot )$ is chosen as the hyperbolic tangent function. Suppose that *K* hops diffusion is considered, and then, the *K*-th power of transition matrix requires an $O(n^2K)$ computational complexity which is prohibited especially for large-scale graphs.

Monti et al. propose a generic graph convolution network framework named MoNet [5] by designing a universe patch operator which integrates the signals within the node neighborhood. In particular, for a node *i* and its neighboring node $j\in \mathcal {N}(i)$, they define a *d*-dimensional pseudo-coordinates $\mathbf{u}(i,j)$ and feed it into *P* learnable kernel functions $\left( w_1(\mathbf{u}),\ldots ,w_P(\mathbf{u})\right)$. Then, the patch operator is formulated as $D_p(i)=\sum _{j\in \mathcal {N}(i)} w_p(\mathbf{u}(i,j)){\mathbf{x}}(j),~p=1,\ldots ,P$, where ${\mathbf{x}}(j)$ is the signal value at the node *j*. The graph convolution in the spatial domain is then based on the patch operator as:

15 $$\begin{aligned} ({\mathbf{x}}*_{s} \mathbf{y})(i) =\sum _{l=1}^{P}\mathbf{g}(p)D_p(i){\mathbf{x}}. \end{aligned}$$

It is shown that by carefully selection of $\mathbf{u}(i,j)$ and the kernel function $w_p(\mathbf{u})$, many existing graph convolutional network models [37, 57] can be viewed as a specific case of MoNet. SplineCNN [58] follows the same framework [i.e., Eq. (15)], but uses a different convolution kernel based on B-splines.

For graphs accompanied with edge attribute information, the weight parameters of filters are often conditioned on the specific edge attributes in the neighborhood of a node. To exploit edge attributes, an edge-conditioned convolution (ECC) operation [59] is designed by borrowing the idea of dynamic filter network [60]. For the edge between node *v* and node *u* at the *p*-th ECC layer, with the corresponding filter-generating network $F^p:~\mathbb {R}^{s}\rightarrow \mathbb {R}^{d_{p+1}\times d_{p}}$ that generates edge-specific weights matrix $\varvec{\Theta }_{v,u}^p$, the convolution operation is mathematically formalized as:

16 $$\begin{aligned} \mathbf{X}^{p+1}(u,:)=\frac{1}{|\mathcal {N}(u)|}\sum _{v\in \mathcal {N}(u)} \varvec{\Theta }_{v,u}^p\mathbf{X}^p(v,:)+\mathbf{b}^p, \end{aligned}$$

where $\mathbf{b}^p$ is a learnable bias and the filtering–generating network $F^p$ is implemented by multi-layer perceptrons.

In addition, Hamilton et al. propose an aggregation-based inductive representation learning model, named GraphSAGE [61]. The full batch version of the algorithm is straightforward: for a node *u*, the convolution layer in GraphSAGE (1) aggregates the representation vectors of all its immediate neighbors in the current layer via some learnable aggregator, (2) concatenates the representation vector of node *u* with its aggregated representation, and then (3) feeds the concatenated vector to a fully connected layer with some nonlinear activation function $\sigma (\cdot )$, followed by a normalization step. Formally, the *p*-th convolutional layer in GraphSAGE contains:

17 $$\begin{aligned}&{\mathbf{x}}_{\mathcal {N}(u)}^{p} \leftarrow \text {AGGREGATE}_{p}(\{\mathbf{X}^p(v,:),~\forall v\in \mathcal {N}(u)\}); \end{aligned}$$

18 $$\begin{aligned}&\mathbf{X}^{p+1}(u,:) \leftarrow \sigma \left( \text {CONCAT}(\mathbf{X}^p(u,:), {\mathbf{x}}_{\mathcal {N}(u)}^{p})\varvec{\Theta }^{p}\right). \end{aligned}$$

There are several choices of the aggregator functions, including the mean aggregator, LSTM aggregator, and the pooling aggregator. By using mean aggregators, Eq. (17) can be simplified to:

$$\begin{aligned} \mathbf{X}^{p+1}(u,:)\leftarrow \sigma \left( \text {MEAN}(\{\mathbf{X}^p(u,:)\}\cup \{\mathbf{X}^p(v,:),~\forall v\in \mathcal {N}(u)\})\varvec{\Theta }^{p}\right), \end{aligned}$$

which approximately resembles the GCN model [37]. Besides, pooling aggregator is formulated as:

$$\begin{aligned} \text {AGGREGATE}_{p}^{\text {pool}}=\max \left( \{\sigma (\mathbf{X}^p(v,:) \varvec{\Theta }^{p}+\mathbf{b}^p),~\forall v\in \mathcal {N}(u)\}\right). \end{aligned}$$

To allow the minibatch training, the authors also provide a variant by uniformly sampling a fixed size of the neighboring nodes for each node [61].

However, the performance in node representation learning is often degraded as the graph convolutional models become deeper. In practice, it has been shown that a two-layer graph convolution model often achieves the best performance in GCN [37] and GraphSAGE [61]. According to [62], the convolution in GCN [37] is related to Laplacian smoothing [63] and more convolution layers result in less distinguishable representations even for nodes from different clusters. From a different perspective, Xu et al. analyze different expansion behaviors for two types of nodes, including the nodes in an expander-like core part and nodes in the tree part of the graphs, and show that the same number of propagation steps can lead to different effects [64]. For example, for nodes within the core part, the influence of their features spreads much faster than the nodes in the tree part and thereby this rapid average causes the node representations indistinguishable. To mitigate this issue and make the graph convolutional models deeper, by borrowing the idea of the residual network [65] in computer vision, Xu et al. propose a skip connection architecture named Jumping Knowledge Network [64]. The Jumping Knowledge Network can adaptively select the aggregations from the different convolution layers. In other words, the last layer of the model can selectively aggregate the intermediate representations for each node independently. The layer-wise aggregators include concatenation aggregator, max-pooling aggregator, and LSTM-attention aggregator. In addition, the Jumping Knowledge Network model admits the combination with the other existing graph neural network models, such as GCN [37], GraphSAGE [61], and GAT [24].

### Related general graph neural networks

Graph convolutional networks that use convolutional aggregations are a special type of the general graph neural networks. Other variants of graph neural networks based on different types of aggregations also exist, such as gated graph neural networks [26] and graph attention networks [24]. In this subsection, we briefly cover some general graph neural network models of which graph convolutional networks can be viewed as special variants.

One of the earliest graph neural networks is [66] which defines the parametric local transition function *f* and local output function *g*. Denote $\mathbf{X}^0(u,:)$ as the input attributes of node *u* and $\mathbf{E}_u$ as the edge attributes of the edges incident to node *u*. Then, the local transition function and local output function are formulated as:

19 $$\begin{aligned}&\mathbf{H}(u,:) =f\left( \mathbf{X}^0(u,:), \mathbf{E}_u, \mathbf{H}(u,:), \mathbf{X}^0(\mathcal {N}(u),:)\right) \end{aligned}$$

20 $$\begin{aligned}&\mathbf{X}(u,:) =g\left( \mathbf{X}^0(u,:), \mathbf{H}(u,:)\right) , \end{aligned}$$

where $\mathbf{H}(u,:)$, $\mathbf{X}(u,:)$ are the hidden state and output representation of node *u*. Eq. (19) defines one general form of aggregations in graph neural network. In [66], the function *f* is restricted to a contraction mapping to ensure convergence and suggested by the Banach’s fixed point theorem [67]. In this way, a classic iterative scheme is applied to update the hidden states. However, it is inefficient and less effective to update the states in an iterative manner to obtain steady states. In contrast, SSE [68] aims to learn the steady states of node representations iteratively but in a stochastic way. Specifically, for a node *u*, SSE first samples a set of nodes $\tilde{\mathcal {V}}$ from $\mathcal {V}$ and updates the node representations for *T* iterations to be close to stability by:

21 $$\begin{aligned} \mathbf{X}(u,:)\leftarrow (1-\alpha )\mathbf{X}(u,:)+\alpha \mathcal {T}_{\varvec{\Theta }} \left[ \{\mathbf{X}(v,:),~\forall v\in \mathcal {N}(u)\}\right], \end{aligned}$$

where node $u\in \tilde{\mathcal {V}}$ and $\mathcal {T}_{\varvec{\Theta }}$ is the aggregation function defined by:

$$\begin{aligned} \mathcal {T}_{\varvec{\Theta }}\left[ \{\mathbf{X}(v,:),~\forall v\in \mathcal {N}(u)\}\right] =\sigma \left( \left[ \mathbf{X}^0(u,:), \sum _{v\in \mathcal {N}(u)}[\mathbf{X}(v,:),\mathbf{X}^0(v,:)]\right] \varvec{\Theta }_2\right) \varvec{\Theta }_1, \end{aligned}$$

where $\mathbf{X}^0(u,:)$ denotes the input attributes of node *u*.

Message-Passing Neural Networks (MPNNs) proposed in [69] generalize many variants of graph neural networks, such as graph convolutional networks (e.g., [37, 56, 61]) and gated graph neural networks [26]. MPNN can be viewed as a two-phase model, including message-passing phase and readout phase. In the message-passing phase, the model runs node aggregations for *P* steps and each step contains the following two functions:

22 $$\begin{aligned} \mathbf{H}^{p+1}(u,:)&=\sum _{v\in \mathcal {N}(u)}M^p(\mathbf{X}^p(u,:),\mathbf{X}^p(v,:), \mathbf{e}_{u,v}) \end{aligned}$$

23 $$\begin{aligned} \mathbf{X}^{p+1}(u,:)&=U^p(\mathbf{X}^p(u,:),\mathbf{H}^{p+1}(u,:)), \end{aligned}$$

where $M^p, U^p$ are the message function and the update function at the *p*th step, respectively, and $\mathbf{e}_{u,v}$ denotes the attributes of edge (*u*, *v*). Then, the readout phase computes the feature vector for the whole graph by:

24 $$\begin{aligned} \hat{\mathbf{y}}=R\left( \{\mathbf{X}^P(u,:)|u\in \mathcal {V}\}\right), \end{aligned}$$

where *R* denotes the readout function.

In addition, Xu et al. theoretically analyze the expressive power of the existing neighborhood aggregation-based graph neural networks [70]. They analyze how powerful the existing graph neural networks are based on the close relationship between graph neural networks and the Weisfeiler–Lehman graph isomorphism test, and conclude that the existing neighborhood aggregation-based graph neural networks (e.g., [37, 61]) can be at most as powerful as the one-dimensional Weisfeiler–Lehman isomorphism test. To achieve the equal expressive power of Weisfeiler–Lehman test, Xu et al. propose a simple architecture named Graph Isomorphism Network [70].

## Applications of graph convolutional networks

Graph convolutional networks can be also categorized according to their application domains. In this section, we mainly introduce the applications of graph convolutional networks in computer vision, natural language processing, science, and other domains.

### Applications in computer vision

Computer vision has been one of the hottest research areas in the past decades. Many existing deep learning architectures used in computer vision problems are built upon the classic convolution neural networks (CNNs). Despite the great successes of CNNs, they are difficult to encode the intrinsic graph structures in the specific learning tasks. In contrast, the graph convolutional networks have been applied to solve some computer vision problems and shown a comparable or even better performance. In this subsection, we further categorize these applications based on the type of data.

#### Images

Image classification is of a great importance in many real-world applications. By some carefully hand-crafted graph construction methods (e.g., kNN similarity graphs) or other supervised approaches, the unstructured images can be converted to the structured graph data and thereby are able to be applied to graph convolutional networks. Existing models for image classification include, but are not limited to [5, 32, 34, 71, 72]. Another application on images is visual question answering that explores the answers to the questions on images. Narasimhan et al. propose a graph convolutional network-based deep learning model to use the information from multiple facts of the images from knowledge bases to aid question answering, which relies less on retrieving the single correct fact of images [73]. In addition, as images often contain multiple objects, understanding the relationships (i.e., visual relationships) among the objects helps to characterize the interactions among them, which makes visual reasoning a hot topic in computer vision. For visual relationships detection, Cui et al. propose a graph convolutional network to leverage both the semantic graphs of words and spatial scene graph [74]. Besides, Yao et al. propose an architecture of graph convolutional networks and LSTM to explore the visual relationships for image captioning [75]. To generate scene graphs, despite some existing message-passing-based methods [76, 77], many of them may not handle the unreliable visual relationships. Yang et al. propose an attentional graph convolutional model that can place attention on the reliable edges while dampening the influence of unlikely edges [78]. In the opposite direction, Johnson et al. use a graph convolutional network model to process the input scene graph and generate the images by a cascaded refinement network [79] trained adversarially [80].

#### Videos

One of the high-impact applications of videos is the action recognition which can help video understanding. In [81], a spatial-temporal graph convolutional model is designed to eliminate the need of hand-crafted part assignment and can achieve a greater expressive power. Another skeleton-based method is [82], where a generalized graph construction process is proposed to capture the variation in the skeleton sequences and the generalized graph is then fed to a graph convolutional network for variation learning. Wang and Gupta [83] represents the input video as a space-time region graph which builds two types of connections (i.e., appearance similarity and spatial-temporal proximity), and then recognizes actions by applying graph convolutional networks. Zhang et al. propose a tensor convolutional network for action recognition [84].

#### Point clouds

Point clouds provide a flexible geometric representation for many applications in computer graphics and computer vision. Followed by the pioneering PointNet [85], the state-of-the-art deep neural networks consider the local features of point clouds [85, 86]. However, these works ignore the geometric relationships among points. EdgeConv [87], on the other hand, is proposed to capture the local geometric structure while maintaining the permutation invariance property and outperforms other existing approaches in the point cloud segmentation task. A regularized graph convolutional network model has been proposed for segmentation on point clouds in [88] in which the graph Laplacian is dynamically updated to capture the connectivity of the learned features. FeaStNet [89] built upon graph convolutional networks dynamically determines the association between filter weights and graph neighborhood, showing a comparable performance in part labeling. Wang et al. propose a local spectral graph convolutional network for both point cloud classification and segmentation [90]. For point cloud classification, other graph convolution-based methods include [45, 59]. Valsesia et al. propose a localized generative model by using graph convolution to generate 3D point clouds [91].

#### Meshes

One application on meshes which we consider in this paper is the shape correspondence, i.e., to find correspondences between collections of 3D shapes. Beyond the classic CNN-based methods (e.g., [92, 93]), several graph convolutional network-based approaches have been proposed, including [5, 89]. In addition, Litany et al. propose to combine graph convolutional networks with variational auto-encoder for the shape completion task [94].

### Applications in natural language processing

Text classification is one of the most classical problems in natural language processing. With the documents as nodes and the citation relationships among them as edges, the citation network can be constructed, in which node attributes are often modeled by the bag-of-words. In this scenario, the straightforward way to classify documents into different categories is by node classification. Many graph convolutional network models have been proposed, to name a few, including [5, 37, 42, 61, 95]. Another way is to view the documents at the graph-level (i.e., each document is modeled as a graph) and classify the texts by graph classification [33, 34]. Besides, TextGCN [96] models a whole corpus to a heterogeneous graph and learn word embedding and document embedding simultaneously, followed by a softmax classifier for text classification. Gao et al. use a graph pooling layer and the hybrid convolutions of graph convolution and classic convolution to incorporate node ordering information, achieving a better performance over the traditional CNN-based and GCN-based methods [97]. When there are lots of labels at different topical granularities, these single-granularity methods may achieve a suboptimal performance. In [98], a graph-of-words is constructed to capture long-distance semantics, and then, a recursively regularized graph convolution model is applied to leverage the hierarchy of labels.

Information extraction is often the cornerstone of many NLP-related applications and graph convolutional networks have been broadly applied in it and its variant problems. For example, GraphIE [99] first uses a recurrent neural network to generate local context-aware hidden representations of words or sentences and then learns non-local dependencies between textual units, followed by a decoder for labeling at the word level. GraphIE can be applied to information extraction such as named entity extraction. Graph convolutional networks have been designed to the relation extraction between words [100, 101] and event extraction [102, 103].

In addition, Marcheggiani et al. develop a syntactic graph convolutional network model that can be used on top of syntactic dependence trees, which is suitable for various NLP applications such as semantic role labeling [104], and neural machine translation [105]. For semantic machine translation, graph convolutional networks can be used to inject a semantic bias into sentence encoders and achieve performance improvements [106]. Moreover, the dilated iterated graph convolutional network model is designed for dependence parsing [107].

### Applications in science

#### Physics

In particle physics, jets are referred to the collimated sprays of energetic hadrons and many tasks are related to jets, including the classification and regression problems associated with the progenitor particles giving rise to the jets. Recently, variants of the message-passing neural network [69] have been designed to classify jets into two classes: quantum chromodynamics-based jets and W-boson-based jets [108]. ParticleNet, built upon edge convolutions [87], is a customized neural network architecture that operates directly on particle clouds for jet tagging [109]. Besides, graph convolutional network model has been also applied for IceCube signal classification [110]. Another interesting application is to predict the physical dynamics, e.g., how a cube deforms as it collides with the ground. Mrowca et al. propose a hierarchical graph-based object representation that decomposes an object into particles and connects particles within the same group, or to the ancestors and descendants [111]. They then propose a hierarchical graph convolutional network to learn the physics predictions.

#### Chemistry, biology, and materials science

Learning on molecules has attracted lots of attention in chemistry, drug discovery, and materials science. For example, graph convolutional networks have been used for molecular fingerprints prediction [56, 112]. In drug discovery, DeepChemStable [113], an attention-based graph convolutional network mode, is used for chemical stability prediction of a compound. Besides, by modeling the protein–protein interactions, drug–protein target interactions into a multimodal graph, graph convolutions can be applied to predict polypharmacy side effects [114]. Another important application in chemistry is the molecular property prediction. Message-Passing Neural Networks (MPNNs) [69], a general graph neural network framework, can be used to predict the quantum properties of a molecular. PotentialNet [115] first entails graph convolutions over chemical bonds to learn the features of atoms, then entails both bond-based and spatial distance-based propagation and finally conducts graph gathering over the ligand atoms, followed by a fully connected layer for molecular property predictions. Protein interface prediction is a challenging problem with important applications in drug discovery. Fout et al. construct a graph where each residue in a protein is considered as a node and nodes are accompanied with features computed from amino acid sequence as well as structure [116]. To predict protein interface, graph convolution layers are used for different protein graphs, followed by one or more fully connected layers. In addition, [117] proposes a so-called crystal graph convolutional neural network to directly learn material properties from the connection of atoms in the crystal.

#### Social network analysis

Beyond the applications in classical problems of social science, such as community detection [42, 118], and link prediction [21, 119, 120], graph convolutional networks have been applied in many other problems. DeepInf [121] aims to predict social influences by learning users latent features. Vijayan et al. propose to use graph convolutional networks for retweet count forcasting [122]. Moreover, fake news can be also detected by graph convolutions [123]. Graph convolutional networks have been widely used for social recommendation which aims to leverage the user–item interactions and/or user–user interactions to boost the recommendation performance. Wu et al. propose a neural influence diffusion model that takes how users are influenced by their trusted friends into considerations for better social recommendations [124]. Ying et al. propose a very efficient graph convolutional network model PinSage[125] based on GraphSAGE [61] which exploits the interactions between pins and boards in Pinterest. Wang et al. propose a neural graph collaborative filtering framework that integrates the user–item interactions into the graph convolutional network and explicitly exploits the collaborative signals [126].

## Challenges and future researches

### Deep graph convolutional networks

Although the initial objective of graph convolutional network models is to leverage the deep architecture for better representation learning, most of the current models still suffer from their shallow structure. For example, GCN [37] in practice only uses two layers and using more graph convolution layers may even hurt the performance. This is also intuitive due to its simple propagation procedure. As deeper the architecture is, the representations of nodes may become smoother even for those nodes that are distinct and far from each other. This issue violates the purpose of using deep models. Although few works have been proposed to address this issue (e.g., skip connection based models), how to build a deep architecture that can better adaptively exploits the deeper structural patterns of graphs is still an open challenge.

### Graph convolutional networks for dynamic graphs

Most of the existing graph convolutional networks explicitly assume the input graphs are static. However, in the real cases, networks are often changing dynamically. For example, social networks are essentially dynamic networks as users are joining/quiting the networks frequently and friendships among users are also changing dynamically. To this end, learning graph convolutional networks on static graphs may not provide an optimal performance. Thus, the efficient dynamic graph convolutional network models are important to be studied.

### More powerful graph convolutional networks

Most of the existing spatial graph convolutional network models are based on neighborhood aggregations. These models have been proved theoretically to be at most as powerful as one-dimensional Weisfeiler–Lehman graph isomorphism test, and the graph isomorphism network has been proposed to reach the limit [70]. However, one natural question to be asked is: can we break the limit of 1-dimensional Weisfeiler–Lehman graph isomorphism test? A few works have studied the related questions such as [127–129]. However, further researches on this problem are still quite challenging.

### Multiple graph convolutional networks

As already mentioned before, the major drawback of the spectral graph convolutional networks is its inability of adaptation from one graph to another graph if two graphs have different Fourier basis (i.e., eigenfunctions of the Laplacian matrix). The existing work [130] alternatively learns the filter parameters by generalizing the eigenfunctions of a single graph to the eigenfunctions of the Kronecker product graph of multiple input graphs. As a different track, inductive learning is possible for many spatial graph convolutional network models, such that one model learned on one or several graphs can be applied to other graphs. However, a drawback of these methods is that the interactions (e.g., anchor links, cross-network node similarities) or correlations (e.g., correlations among multiple views) across multiple graphs are not exploited. In fact, given multiple graphs, the representation learning of a unique node should be able to benefit from more information provided across graphs or views. However, to our best knowledge, there is no existing model aiming at the problems in this setting.

## Concluding remarks

Graph convolutional network models, as one category of the graph neural network models, have become a very hot topic in both machine learning and other related areas, and a substantial amount of models have been proposed to solve different problems. In this survey, we conduct a comprehensive literature review on the emerging field of graph convolutional networks. Specifically, we introduce two intuitive taxonomies to group the existing works based on the types of graph filtering operations and also on the areas of applications. For each taxonomy, we highlight with some detailed examples from a unique standpoint. We also discuss some open challenges and potential issues of the existing graph convolutional networks and provide some future directions.

## Data Availability

Not applicable.

## Abbreviations

CNN: convolution neural network
ChebNet: Chebyshev polynomial based graph convolution (model proposed in [34])
GCN: graph convolutional network (model proposed in [37])
FastGCN: minibatch training for GCN (model proposed in [39])
CayleyNet: Cayley polynomial based graph convolution (model proposed in [42])
LanczosNet: multiscale graph convolutional network by Lanczos algorithm (model proposed in [43])
PATCHY-SAN: graph CNN (model proposed in [52])
LGCN: large-scale graph CNN (model proposed in [53])
DCNN: diffusion-based graph convolutional network (model proposed in [57])
MoNet: pseudo-coordinates based graph convolutional network (model proposed in [5])
SplineCNN: B-splines based convolution kernel for graphs and meshes (model proposed in [58])
ECC: edge-conditioned convolution (model proposed in [59])
GraphSAGE: mean/LSTM/pooling aggregation based graph convolutional network (model proposed in [61])
GAT: graph attention network (model proposed in [24])
MPNN: message passing neural networks (model proposed in [69])

## Footnotes

1 The symbol *i* here represents an imaginary number, instead of an index using in other parts of this paper.
